# A novel edge based embedding in medical images based on unique key generated using sudoku puzzle design

**DOI:** 10.1186/s40064-016-3356-1

**Published:** 2016-09-29

**Authors:** B. Santhi, B. Dheeptha

**Affiliations:** Department of Information and Communication Technology, School of Computing, SASTRA University, Thirumalaisamudram, Thanjavur, 613401 India

**Keywords:** Telemedicine, Edge based embedding, XOR coding, Stego image, Least significant bit

## Abstract

The field of telemedicine has gained immense momentum, owing to the need for transmitting patients’ information securely. This paper puts forth a unique method for embedding data in medical images. It is based on edge based embedding and XOR coding. The algorithm proposes a novel key generation technique by utilizing the design of a sudoku puzzle to enhance the security of the transmitted message. The edge blocks of the cover image alone, are utilized to embed the payloads. The least significant bit of the pixel values are changed by XOR coding depending on the data to be embedded and the key generated. Hence the distortion in the stego image is minimized and the information is retrieved accurately. Data is embedded in the RGB planes of the cover image, thus increasing its embedding capacity. Several measures including peak signal noise ratio (PSNR), mean square error (MSE), universal image quality index (UIQI) and correlation coefficient (R) are the image quality measures that have been used to analyze the quality of the stego image. It is evident from the results that the proposed technique outperforms the former methodologies.

## Background

Information security has always been a matter of great concern with cyber threats on the rise. It is a vital aspect, especially in the field of telemedicine. Telemedicine utilizes telecommunication and information technology to provide health care. In such scenarios, the patient’s data must stay confidential, failing of which could result in a huge complication.

Steganography offers a viable solution to protect confidential information from unauthorized access by concealing the existence of data (Lou and Liu 2002). It is an art of hiding data inside any cover object (Amin et al. 2003). The cover object could be an image, a video or an audio file. The most commonly preferred cover object is an image. Different steganographic algorithms exist for different image formats (Morkel et al. 2005). Images can be either be a color image, or a grey image, or a binary image. The color images can again be in various formats like hue saturation value (HSV), luminance chrominance (YCbCr), red green blue(RGB), YUV, YIQ, etc.

**Fig. 1:**
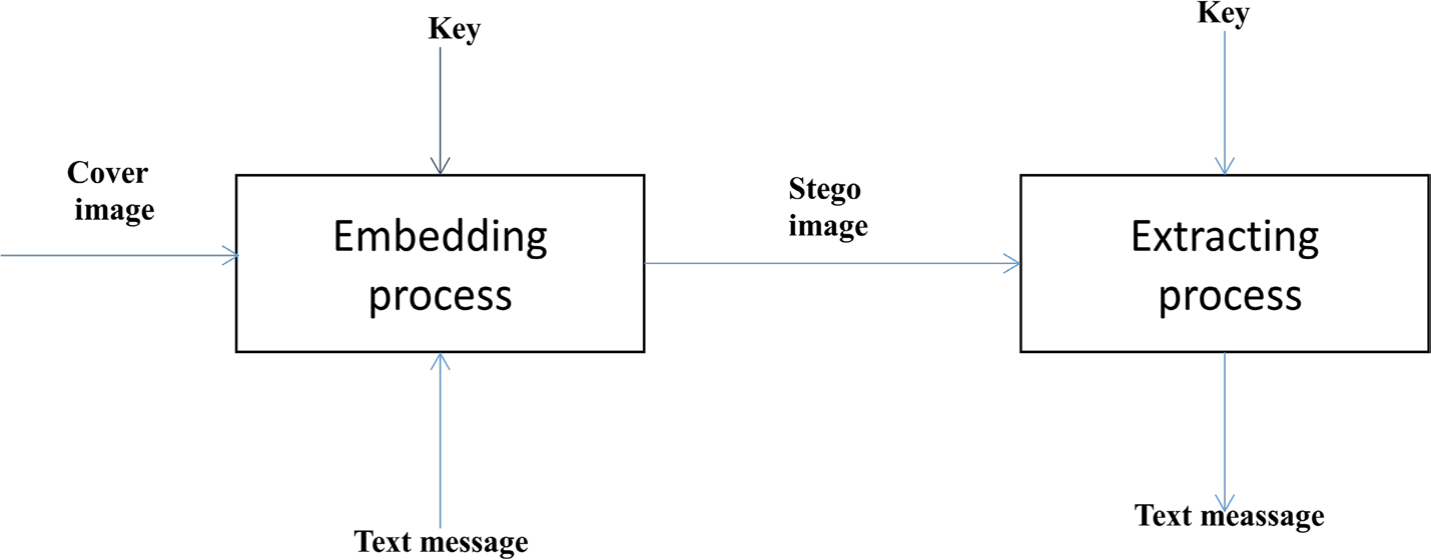
Block representation of the embedding phase and the extraction phase

If C is the cover image and S is the stego image, the embedding process is represented as S = EM(C, M, K), where M is the message and K is the key used and EM is the embedding function. Similarly, the extraction process is defined as M = EX(S, K). The embedding phase and the extraction phase are represented as shown in Fig. 1.

**Fig. 2:**
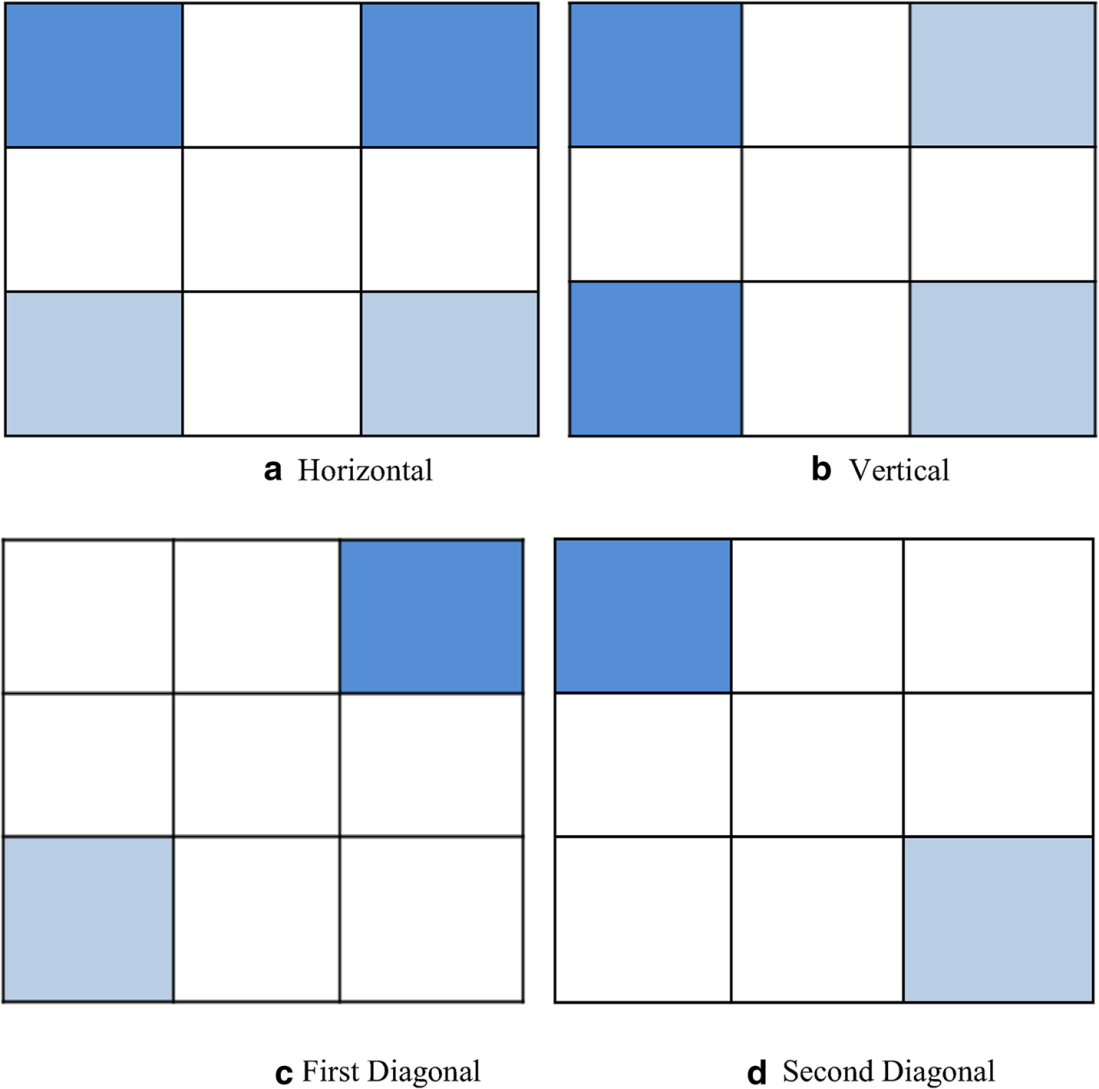
3 × 3 block edges magnitude

**Fig. 3:**
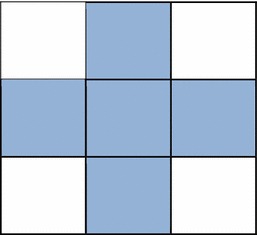
Selected pixels for embedding in a 3 × 3 edge block

A plethora of steganography techniques have been suggested in the erstwhile research works (Cheddad 2010). They can be broadly categorized on the basis of the embedding domain namely, spatial and frequency domain (transform domain). In the case of spatial domain, M is directly embedded in the pixels of C using the least significant bit (Canny 1986) or pixel value differencing (PVD). In the transform domain, many transforms are applied to the base image and M is embedded by altering the coefficient values obtained from transformation. The most commonly used transforms include DWT (discrete wavelet transform), SVD (singular value decomposition) and DCT (discrete cosine transform).

The steganography method used must offer imperceptibility, capacity, security and robustness (Cheddad 2010). The variations in the embedded image must be subtle enough to be inconspicuous to the human eye and have a good imperceptibility. This is achieved by utilizing the LSB of the pixels. This also facilitates easy retrieval of the embedded data. Coding methods like matrix coding, XOR coding etc., are incorporated to enhance the embedding efficiency. Generally, a random number is generated to improve the security of the algorithm. This also ensures that the key changes for every session.

This paper proposes a steganography method in the spatial domain for an RGB image. This method identifies the edge blocks of the cover image and combines with it an XOR coding function. It also generates a unique key for every session, thus ensuring robustness of the technique.

The remaining paper is structured in the following manner—"Related works" section briefs about the existing research works, analyzing their strengths and drawbacks. The detailed elaboration of the proposed methodology is elicited in "The proposed method" section along with the necessary flow charts. The experimental results are analyzed in "Experimental results and discussions" section and "Conclusion" section wraps up the entire paper work.

## Related works

This section briefs about the various methodologies that are used to perform steganalysis. Steganography methods can use any form of cover object to embed message bits. Cover objects include image, video and audio files. A video steganography algorithm was proposed by Mritha and Ashidi (2015) in which data is concealed in a video. This paper has succeeded in reducing the distortion in the cover video and securely embedding the message using scene-change detection. Similarly, an audio steganography scheme was proposed by Kar and Mulkey (2015) in 2015 which uses multiple threshold values to restrict the number of bits embedded in the sample audio.

This paper has classified the steganography techniques based on the domain (Spatial or transform) used for implementation. In 2004, Chan and Chen (2004) presented a data hiding technique in spatial domain using plain LSB substitution. Optimal pixel adjustment process was applied to the stego image to improve its quality. However, such simple LSB substitution methods are easily prone to attacks. Another spatial domain technique that is often used is based on pixel value differencing(PVD).This was proposed by Da-Chun Wu and Wen-Hsiang Tsai (2003) in 2003. The message was embedded based on the differences obtained between two consecutive pixels. The range of difference values chosen depends on the human vision’s sensitivity to variations in the image. Although PVD can be used to conceal a large number of secret bits, the histogram plotted for pixel differences can expose the presence of the secret message. To overcome such anomalies, Zhang and Wang (2003) presented a modified version of the method in 2004 wherein a pseudo random number generated from an embedding key was used for any pair of successive pixels.

Another alternative advancement to the PVD technique was the utilization of the edge pixels to embed message bits. Edge blocks are employed in accordance to the human visual system since human eyes are more sensitive to changes in smooth areas rather than sharp, contrast regions. The intensity of edge pixels was either higher or lower than their neighboring pixels, thus causing a sharp variation in the image. Hence edge blocks are most suitable to hide secret information in an image. Numerous edge detection methods have been elicited over the years. In 1986, Canny (1986) proposed a computational approach to edge detection which involved satisfying a few localization conditions, detection and response criteria on a class of edges. Li et al. (2009) used sobel operator to generate edge image. Edge detection was performed on all the three (R, G, B) planes and the corresponding LSB of each pixel are utilized to embed data. Finally, the stego planes were merged to obtain the stego image. But, this method did not ensure high embedding capacity. Chen et al. (2010) introduced a hybrid edge detector by combining fuzzy edge detector and the canny edge detector, thus effectively achieving a good quality stego image. However, this method brings about unnecessary modifications in the stego image.

Bassil (2012) proposed an image steganography for color images based on canny edge detection in 2012. Three least significant bits of every edge pixel identified by the canny edge detector are replaced by message bits. In addition to this, the algorithm is characterized by three parameters that help to yield different outputs for the same image and data. However, it does not guarantee the correct retrieval of the hidden message. In 2013, Modi et al. (2013) applied canny edge detection to color images. The least two significant bits of every edge pixel are used for embedding data. Edges are selected depending on the length of the secret message to be embedded.

All the mentioned works utilized canny edge detector which, unfortunately, does not produce the same set of edge pixels for a pair of cover image and stego image. Consequently, the message extracted could turn out to be incorrect. To evade from such inconsistencies, Dmour and Ani (2016) proposed an embedding technique using edge based detection and XOR coding. The cover image is broken down into non-overlapping blocks. The edge blocks of the cover image and the stego image are identified using a threshold value. The current paper is an improvisation of Dmour’s work to improve the security of the algorithm by generating an exclusive key matrix for every session.

To augment the embedding efficiency, a myriad of coding theories have been proposed. They include matrix encoding, chaotic sequencing and parity bit check. In 2007, Liu and Xi (2007) used chaotic sequencing concept for information hiding. In 1998, Crandall et al. (1998) applied matrix embedding to minimize the disparities between the original image and the stego image. XOR operation is used to hide two message bits in a block of three pixels. F5 algorithm, a transform domain implementation was then introduced by Westfeld (2001) in 2001. This algorithm serves two purposes—improving embedding efficiency and minimizing the change of DCT coefficients. K bits of secret data is concealed in 2^*k*^*−*1 cover bits using hamming code. Despite the improvements in embedding efficiency, matrix encoding has certain drawbacks such as, limited embedding capacity and high computational cost. Hou et al. (2011) proposed a tree based parity check approach that utilizes a tree structure to minimize the distortions in the cover image. This method can hide 2^n^ bits of message bits in n-level binary tree.

This paper proposes an embedding technique based on XOR coding. XOR coding reduces the computational cost to a great extent and also helps in easy retrieval of the message bits.

## The proposed method

### Identification of edges

The human eyes are less affected by changes in image regions containing edges and sharp transitions in comparison to smooth regions. Consequently, the message is camouflaged in the edge areas to render the steganography algorithm imperceptible.

The gamut of traditional edge detection algorithms described earlier result in edge images that are susceptible to changes in the original cover image, in spite of the changes being minor or insignificant. The common edge detection algorithms that are in use include Sobel, Canny, Prewitt, Roberts and fuzzy logic methods. Since the concealment of information in edge images generated by these methods would result in changes to the cover image, the algorithm proposed in (Al-Dmour 2016) has been adopted.

The method given by Al-Dmour (2016) identifies the edge images such that the edge images of the cover image and the stego image remain identical. This assures the correct retrieval of the embedded message. The algorithm divides the cover image into non-overlapping blocks that are individually analyzed for inclusion as edge blocks. The detailed steps are as follows:

**Table:** 

Algorithm: Edge detection	
Input:	C, a cover image
	th, a threshold value between 4 and 96. Choose the highest value of 96 initially
Output:	E, an edge image.
1. Divide C into non-overlapping blocks, each of size 3 × 3	
2. Compute the absolute mean difference between the left and right columns of the block. Repeat the same to find the horizontal, first diagonal and the second diagonal’s magnitude. Figure 2 depicts this calculation	
3. The maximum of the four values is assigned to a variable e. If e > th, the block is considered to be an edge block. Else, the block is a non-edge block	
4. Construct a matrix E with the value e for edge blocks and a value of 0 for non-edge blocks	
5. The message is embedded only in the edge blocks as shown in Fig. 3	

Only the five of the nine pixels of the identified edge block are used for hiding the secret message. The remaining four pixels are not disturbed since they are used in estimating the edge strength. This ensures that the edge strength of the blocks in the cover image and stego image remains unchanged.

### Message embedding

**Fig. 4:**
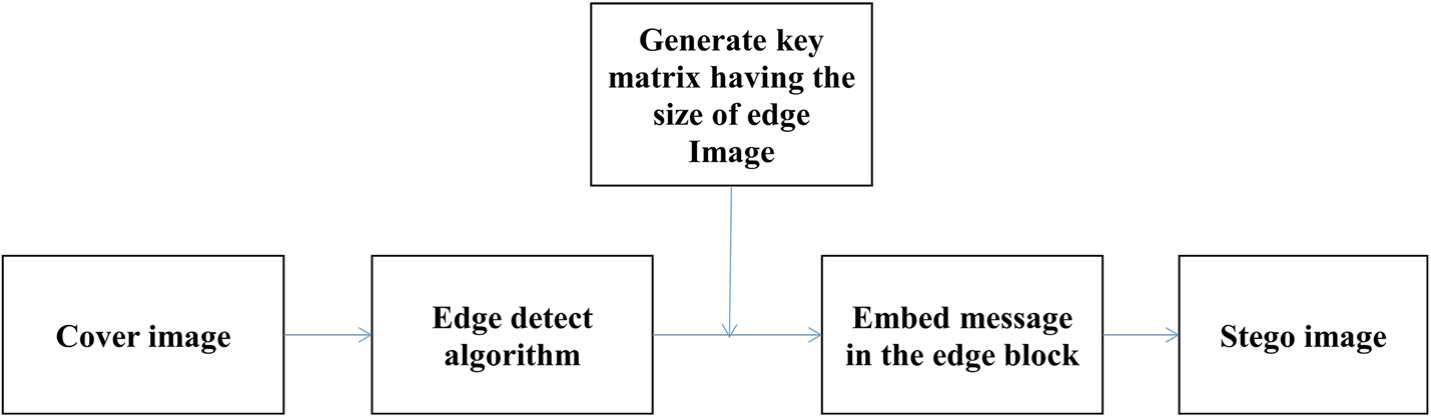
Message embedding process

The embedding process in the spatial domain is depicted in Fig. 4 as a flowchart. Firstly, the medical cover image and the secret message are read. Only one bit is embedded in each pixel. The threshold value is initially set to the highest value, which is 96 according to the work in Al-Dmour (2016). The threshold value is then adjusted depending on the number of pixels required, length of message according to the following condition:

No. of edge pixels $\ge \frac{{4 * {\text{message length}}}}{3}$

The secret message bits are hidden in the discovered edge pixels using XOR coding and the unique key generated. The key matrix is of the same size as E, the edge image. The message bits are concealed in an edge block only if the corresponding value for the edge block in the key matrix is either 1 or 9. This helps in randomizing the selection of edge blocks, thus enhancing the security of the algorithm. The LSBs of the edge pixels are split into groups of four each. Three message bits are embedded into the pixels of each group using XOR operation. XOR operation guarantees that only a minimum number of pixels change. For instance, message bits MP_1_, MP_2_ and MP_3_ are embedded in the last bit of the first edge pixel group P_1_, P_2_, P_3_ and P_4_ in the following manner:

**Table:** 

Algorithm: Message embedding
1. The three key values to be compared with the message bits are computed as follows: KP_1_ = P_1_ XOR P_2_ KP_2_ = P_3_ XOR P_4_ KP_3_ = P_1_ XOR P_3_
2. The obtained values of KP_1_, KP_2_, KP_3_ are compared with MP_1_, MP_2_, MP_3_. The comparisons result in either of the eight conditions, as mentioned in Table 1. Let the new bits obtained be QP_1_, QP_2_, QP_3_ and QP_4_ of the stego image. On an average, this comparison results in a modification of 1.25 bits
3. The threshold value is also embedded in the cover image since it will be required during retrieval process. Conventionally, the threshold value is embedded in the last pixel of the cover image

### Message extraction

**Fig. 5:**
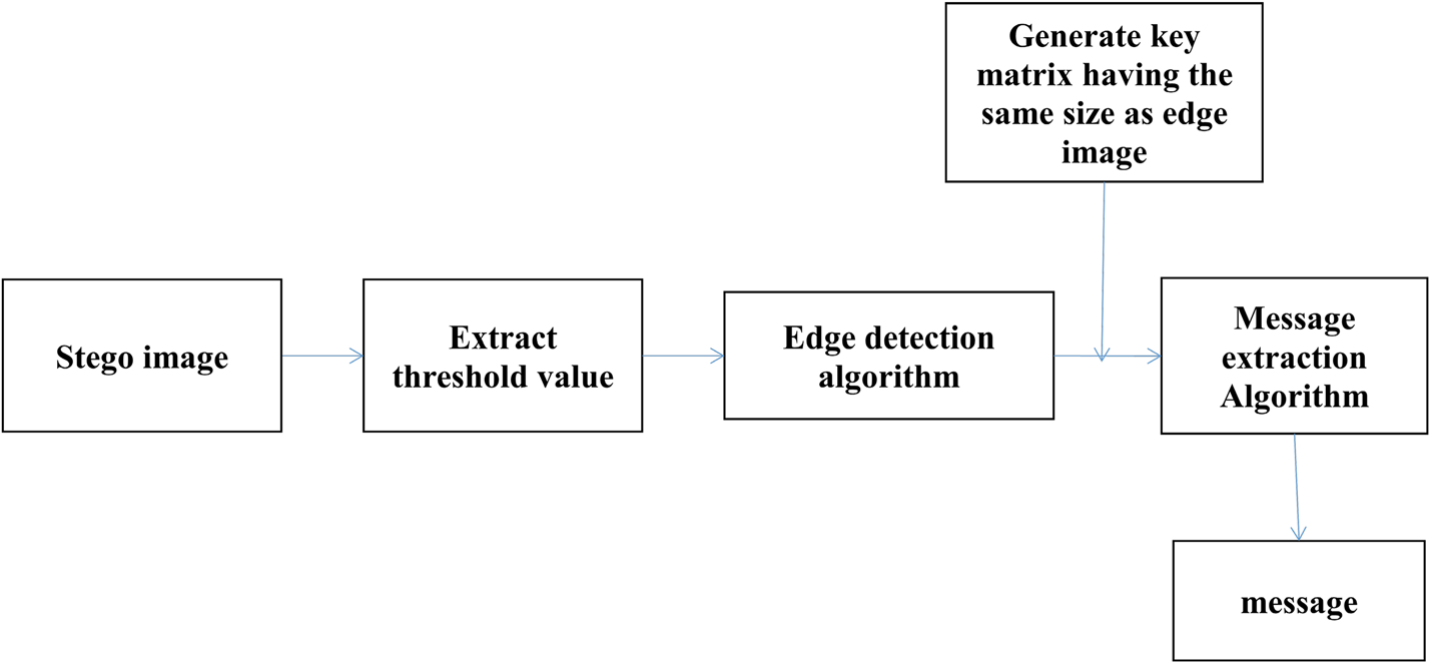
Message extraction process

Figure 5 shows the flowchart for the message extraction process in the spatial domain. This is a relatively quicker and an effortless process than the embedding process. Firstly, the threshold value is retrieved. The edge image of the stego image is obtained using the retrieved threshold. This edge image should be the same as the one obtained in the embedding stage. Similar to the embedding stage, the next step is to divide the LSBs of the edge blocks into groups of four. The same key matrix is then generated. The following operations are performed on the edge blocks whose corresponding values in the key matrix are either 1 or 9:

For all sets of four stego edge bits QP_1_, QP_2_, QP_3_ and QP_4_, the message bits MP_1_, MP_2_ and MP_3_ are retrieved thus:

MP_1_ = QP_1_ XOR QP_2_

MP_2_ = QP_3_ XOR QP_4_

MP_3_ = QP_1_ XOR QP_3_

These equations guarantee the correct result for any combination of message bits and edge bits. Thus the message is accurately reinstated.

### Key generation

**Fig. 6:**
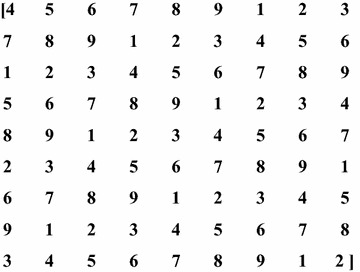
Key matrix used in embedding and extraction process

The design of a sudoku puzzle is taken as the template to generate the required key. The size of the key must be the same as that of E, the edge image. A random number between 1 and 9 is chosen as the first element of the key matrix. Initially, a 9 × 9 block is generated such that all the nine numbers (1 to 9) are present along any row, column and in the non-overlapping 3 × 3 sub blocks. A random number is chosen as the initial element so that the key matrix changes for every session. The key matrix for one such session is given in Fig. 6 with the initial element being 4.

This 9 × 9 block is replicated along the rows and columns to obtain the final key matrix whose dimensions are the same as that of the edge image, E. Suppose the edge image has a size of 81 × 81. The 9 × 9 block has to be replicated nine times along the row and nine times along the column to produce the final key matrix.

This kind of key generation has been adopted to enhance the security of the algorithm. Firstly, the entire key need not be passed to the embedding or the extracting algorithm. It would be adequate to pass the first element of the key matrix to the two algorithms for efficient operation. The matrix can then be generated as described above. Besides, the inclusion of the key matrix in this algorithm helps in randomizing the selection of the edge blocks for embedding the message bits. In this paper, when a key matrix value of 1 or 9 is encountered, the edge block is utilized for hiding the message bits. In the absence of the key matrix, the edge blocks are selected sequentially to embed information. This might increase the probability of attacks.

### Improving the embedding capacity

To improve the embedding capacity of the cover image, all the three planes namely, the red plane, the blue plane and the green plane of the RGB image are utilised to conceal the payload. The edge image and the key matrix are obtained for each plane using the algorithms in the proposed algorithm. The threshold value may vary for each plane. Hence, the threshold value is embedded in the last pixel of each plane. The message bits are embedded in the three planes in the order of red, green and blue.

In the retrieval process, the edge images and the key matrix for the stego image are generated for each plane. The XOR operations are then applied to extract three message bits at a time and finally the entire message is restored.

### An example

**Fig. 7:**
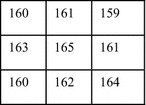
A 3 × 3 block of the input image

The following section illustrates the working of the edge based embedding algorithm using a 3 × 3 block as shown in Fig. 7. Suppose that the threshold value is 3.

Here, the output of edge detection algorithm is greater than the threshold value. Hence the block is an edge block and suitable for embedding. At this stage, it is ensured that the corresponding key matrix value for the edge block is either 1 or 9.

Consider the same block as given in Fig. 7. According to the embedding algorithm, three message bits can be embedded in four edge pixels.

From Table 1, it is apparent that the sixth condition satisfies. Hence P_1_ is complemented. P_1_ = 0.

**Table 1:** Embedding conditions

Condition			Action to be taken
MP_1_ = KP_1_	MP_2_ = KP_2_	MP_3_ = KP_3_	No change
MP_1_ = KP_1_	MP_2_ = KP_2_	MP_3_! = KP_3_	Complement P_3_, P_4_
MP_1_ = KP_1_	MP_2_! = KP_2_	MP_3_ = KP_3_	Complement P_4_
MP_1_ = KP_1_	MP_2_! = KP_2_	MP_3_! = KP_3_	Complement P_3_
MP_1_! = KP_1_	MP_2_ = KP_2_	MP_3_ = KP_3_	Complement P_2_
MP_1_! = KP_1_	MP_2_ = KP_2_	MP_3_! = KP_3_	Complement P_1_
MP_1_! = KP_1_	MP_2_! = KP_2_	MP_3_ = KP_3_	Complement P_2_, P_4_
MP_1_! = KP_1_	MP_2_! = KP_2_	MP_3_! = KP_3_	Complement P_1_, P_4_

Thus the first pixel value of the group becomes 160 and the other pixel values remain the same.

They are the exact message bits that were initially embedded. Thus the message is reinstated successfully.

## Experimental results and discussions

Conventionally Zhou (2004), three standard tests are carried out to analyze the efficiency of any steganography algorithm. The first test is used to evaluate the embedding efficiency (change rate) of the stego image; the second one is used to assess the embedding payload of the cover image and lastly the security level of the proposed technique is determined. The proposed method has been implemented in MATLAB R2013a. An RGB image of size 246 × 246 × 3 has been used for implementing the algorithm. This paper exploits the red plane for all the operations on the image. Hence all the measures have been described in terms of two-dimensional images. The method can be extended to the other planes to increase the embedding capacity. This section presents the results and analysis of the proposed method.

### Embedding capacity evaluation

**Fig. 8:**
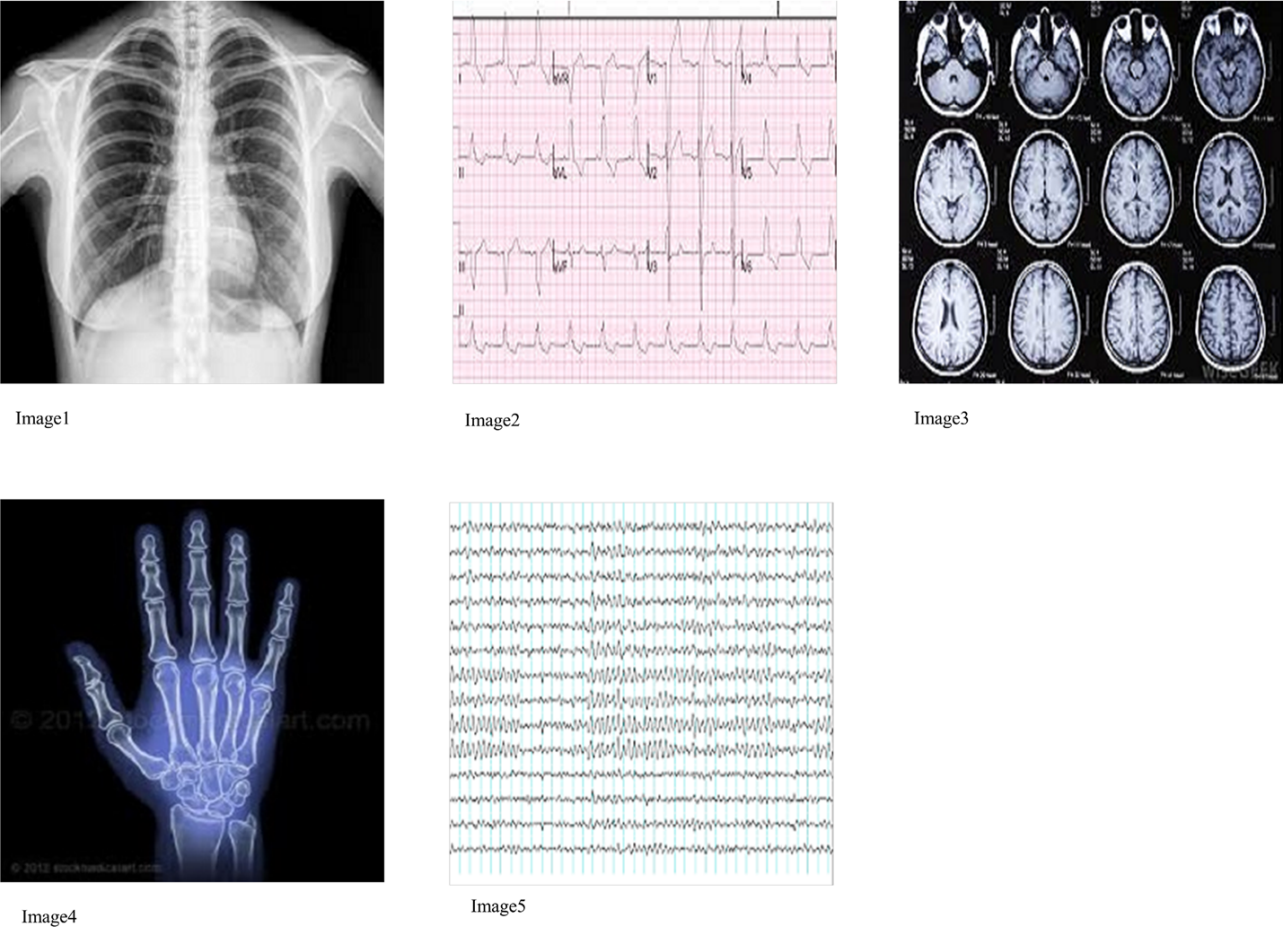
Cover images

Embedding capacity defines the number of bits that can be embedded in the cover image. A high value of embedding capacity is an appreciable characteristic. It is computed using Eq. 1.

1 $${\text{E}} = \frac{K}{WH}$$

where K is the maximum number of secret message bits that can be embedded in the image of size H × W. The embedding rate depends on the cover image size and the threshold value used to identify edges. Table 2 summarizes the embedding capacity of the five images shown in Fig. 8. used in this experiment. The embedding rates are generally the same for all images in LSB-based embedding algorithms. However, the embedding capacity of each image vary depending on the threshold value in this experiment

**Table 2:** Embedding capacity

Image	Embedding capacity
Image1	0.0146
Image2	0.0286
Image3	0.1585
Image4	0.0279
Image5	0.0931

### Embedding distortion evaluation

A few standard measures exist to compute the quality of the stego image. Some of these include PSNR, MSE, SSIM and correlation coefficient(R). These measures analyze the stego image by comparing it with the original cover image. Table 3 gives the equations used to evaluate the measures.

**Table 3:** Image quality measures

Measures	Formula
PSNR	10log_10_ $\left( {\frac{256*256}{MSE}} \right)$
MSE	$\frac{{\mathop \sum \nolimits_{{{\text{i}} = 1}}^{\text{m}} \mathop \sum \nolimits_{{{\text{j}} = 1}}^{\text{n}} ({\text{A}}\left( {{\text{i}},{\text{j}}} \right) - {\text{B}}({\text{i}},{\text{j}}))^{2} }}{m*n}$
SSIM	$\frac{{(2 * {{\upmu }}_{\text{A}} * {{\upmu }}_{\text{B}} + {\text{c}}_{1} ) (2 * {{\upsigma }}_{\text{AB}} + {\text{c}}_{2} ) }}{{ ({{\upmu }}_{\text{A}}^{2} + {{\upmu }}_{\text{B}}^{2} + {\text{c}}_{1} )({{\upsigma }}_{\text{A}}^{2} + {{\upsigma }}_{\text{B}}^{2} + {\text{c}}_{2} )}}$
UIQI	$\frac{{4 * {{\upsigma }}_{\text{AB}} * {{\upmu }}_{\text{A}} * {{\upmu }}_{\text{B}} }}{{({{\upsigma }}_{\text{A}}^{2} + {{\upsigma }}_{\text{B}}^{2} )({{\upmu }}_{\text{A}}^{2} + {{\upmu }}_{\text{B}}^{2} ) }}$
R	$\frac{{\mathop \sum \nolimits_{{{\text{i}} = 1}}^{\text{N}} \left( {\frac{{{\text{A}}_{\text{i}} - {{\upmu }}_{\text{A}} }}{{{{\upsigma }}_{\text{A}} }}} \right)\left( { \frac{{{\text{B}}_{\text{i}} - {{\upmu }}_{\text{B}} }}{{{{\upsigma }}_{\text{B}} }}} \right)}}{{{\text{N}} - 1}}$

The peak signal to noise ratio is evaluated using the equation mentioned in Table 2. MSE is the mean square error between the cover image A and the stego image B, each of size m × n.

A higher value of PSNR and a lower value of MSE signify a better quality.

The structural similarity index metric (SSIM) evaluates the visual impact of three characteristics of an image- luminance, contrast and structure. Structural information reveals that pixels that are spatially close have stronger interdependencies.

$\mu_{\text{A}} , \, \mu_{\text{B}} ,\;\sigma_{A} ,\;\sigma_{B}$ in the equation are the local means, standard deviations of images A, B, and σ_AB_ is the cross variance of A and B.

c_1_ = (L_1_X)^2^ and c_2_ = (L_2_X)^2^ are two constants used to avoid null denominator.

X is the dynamic range and is typically equal to (2^no. of bits per pixel^ − 1), where L_1_ = 0.01 and L_2_ = 0.03.

The range of SSIM is between –1 and 1. Identical images yield a maximum value of 1.

Universal image quality index (UIQI) is also used as an image quality measure (Wang 2002). It is a product of three factors: loss of correlation, contrast distortion and luminance distortion.

μ_A_, μ_B_, σ_A_^2^,  σ_B_^2^ in the equation are the mean values and variance of images A and B respectively.

The dynamic range of UIQI is between −1 and 1. A value of 1 is obtained for identical images.

The correlation coefficient (R) is also an essential measure to predict the quality of the stego image. μ_A_ and σ_A_ in the equation are the mean and standard deviation of A, respectively, and μ_B_ and σ_B_ are the mean and standard deviation of B, respectively.

**Fig. 9:**
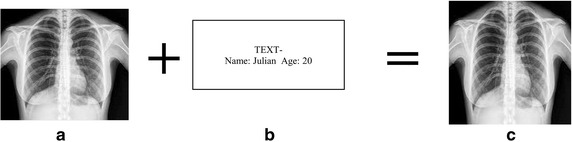
**a** Original image **b** message to be embedded **c** Stego image

Figure 8 illustrates the five different cover images used in this experiment. A sample cover image along with the text to be embedded is shown in Fig. 9. The obtained stego image is also shown. Further results have been drawn using the same text message.

**Fig. 10:**
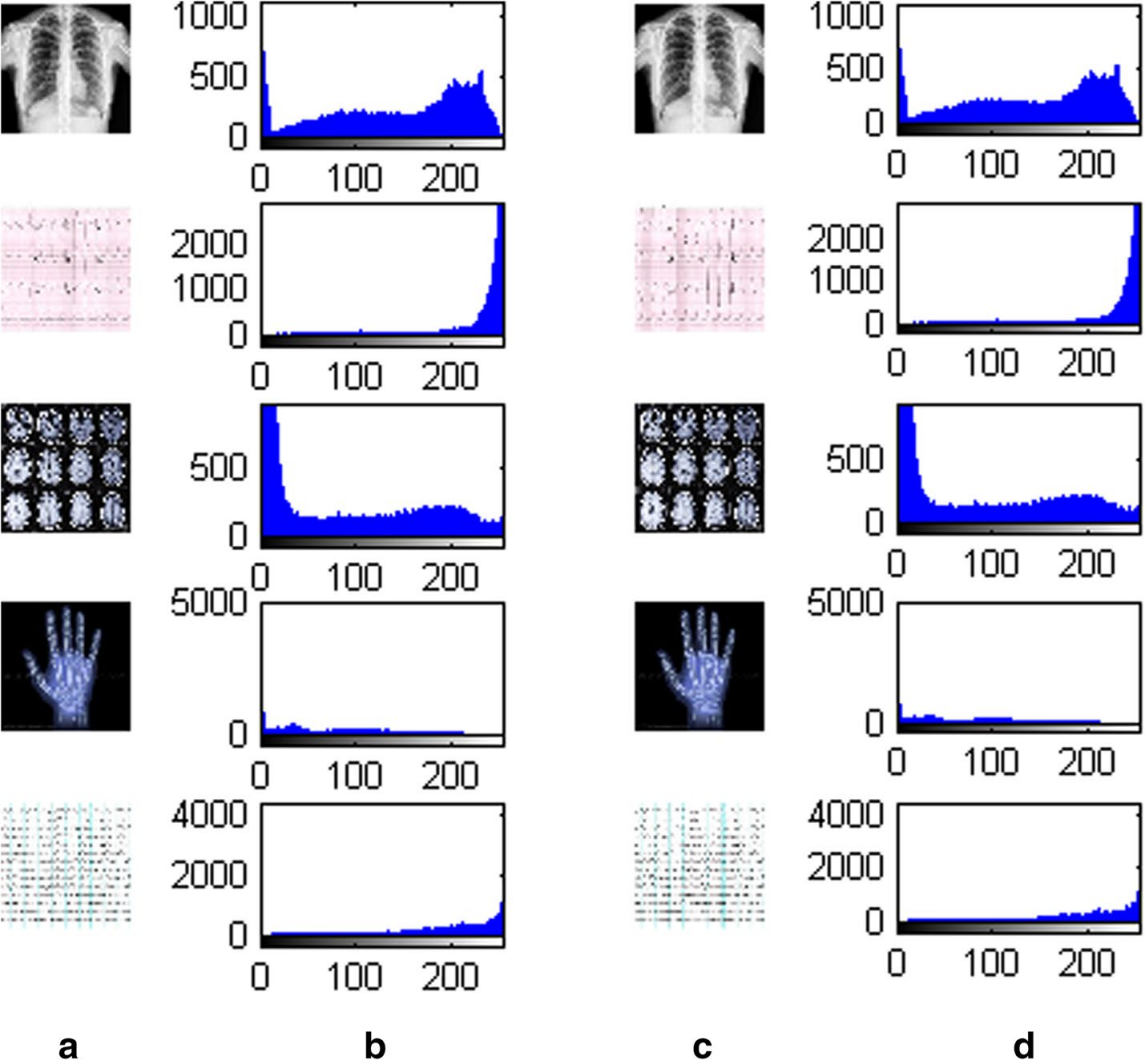
**a** Cover images **b** histogram of the cover images **c** corresponding stego images and **d** histogram of the stego images

**Fig. 11:**
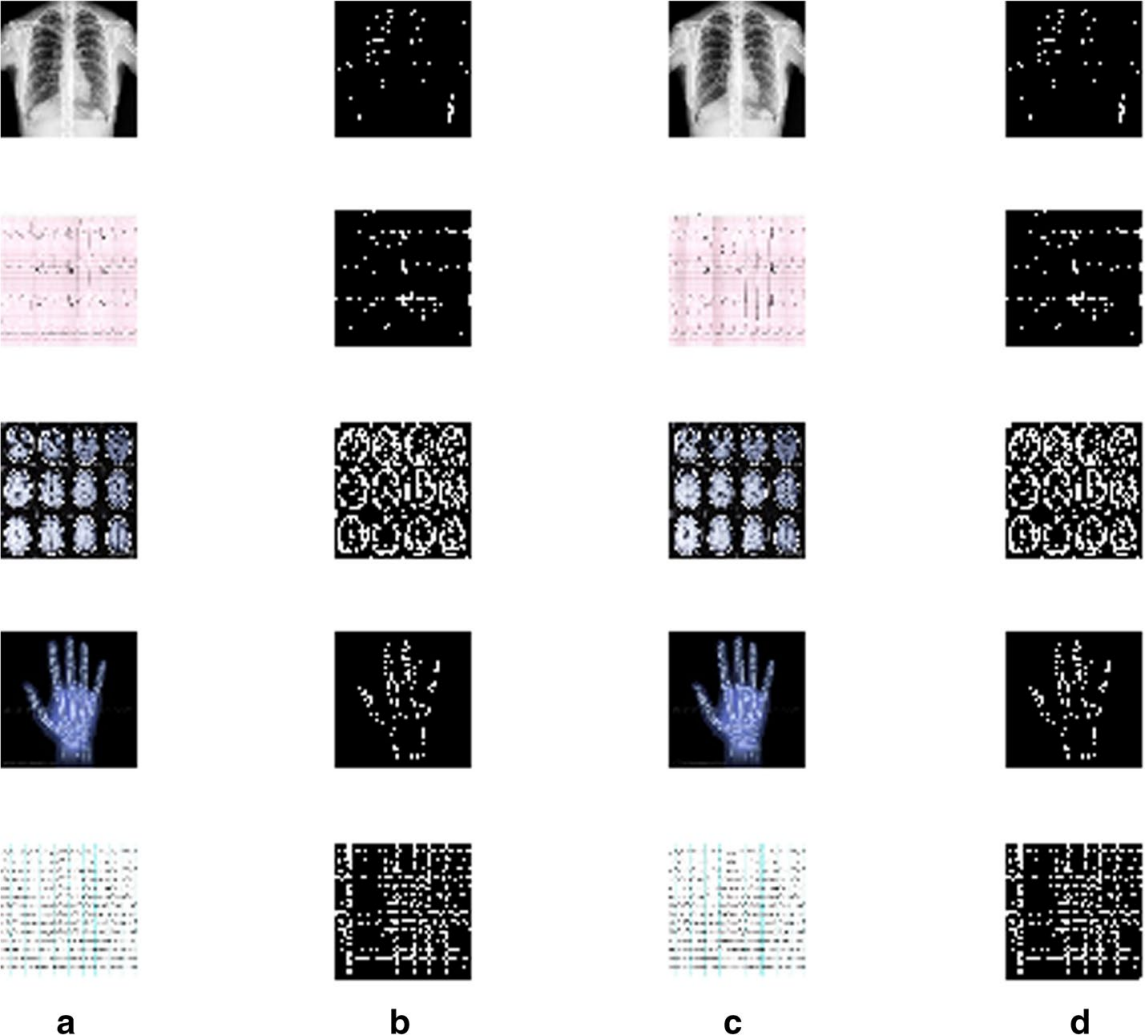
**a** Cover image **b** cover edge image **c** stego image and **d** stego edge image

Figure 10 shows the histograms of the cover images and the stego images. It is observed that there exist no obvious differences between the histograms of the cover image and the stego image. Similarly, Fig. 11 shows the edge images of the various cover images and the stego image. There is high degree of similarity between the cover edge image and the stego edge image.

Table 4 gives the values of the various image quality measures applied to the five different images, given the same embedding capacity. The comparison of the proffered method and Al-Dmour’s method is presented in Table 5. The embedding capacity of the images remain equal in both the methods. Table 6 compares the cover edge image and the stego edge image using the same measures. The results indicate that the proposed method surpasses the former method.

**Table 4:** The image quality measures of five images for the same embedding capacity

Cover image 246 × 246 × 3	PSNR in dB	MSE	UIQI	R
Image1	80.9320	5.2879e−04	1.0	1.0
Image2	71.3896	0.0048	1.0	0.9995
Image3	80.6687	5.6183e−04	1.0	1.0
Image4	80.5428	5.7836e−04	1.0	1.0
Image5	71.2268	0.0049	1.0	1.0

**Table 5:** Comparison of the proposed method with the Al-Dmour’s method using PSNR and MSE values

Image	Proposed method		Hayat Al-Dmour & Ahmed Al-Ani’s method	
	PSNR	MSE	PSNR	MSE
Image1	80.9320	5.2879e−04	80.6687	5.6183e−04
Image2	71.3896	0.0045	71.3595	0.0048
Image3	80.6687	2.3134e−04	80.1857	6.2793e−04
Image4	80.5428	1.9829e−04	80.4205	5.9488e−04
Image5	71.2268	0.0046	71.1834	0.0050

**Table 6:** Image quality measures of the edge images

Cover image 246 × 246	PSNR in dB	MSE	UIQI	R
Image1	∞	0	1.0	1.0
Image2	95.9835	1.6525e−05	0.9985	0.9985
Image3	∞	0	1.0	1.0
Image4	∞	0	1.0	1.0
Image5	95.9835	1.6525e−05	0.9995	0.9995

We observe an increase of 0.327, 0.042, 0.602, 0.152 and 0.06 % in the PSNR values of Image1, image2, image3, image4, image5, respectively, using the proposed method. A higher PSNR value indicates minimized distortions in the cover image. This analysis suggests that the proposed method is superior to the former method (Al-Dmour 2016).

The above table indicates that there are no differences between the cover edge images and the stego edge images for Image1, Image3, Image4 and very slight differences in Image2 and Image5. This eventually proves that the edge detection algorithm adopted is the most effective algorithm implemented till date.

## Conclusion

This paper presents a robust steganography method which conceals patients’ information in the edge blocks of any medical image like X-ray or EEG or ECG. It exploits the fact that human eyes are less liable to changes in sharp regions of the image and hence uses the high contrast edge pixels to embed data, thereby minimizing the distortions in the stego image. The embedding capacity is improved by extending the algorithm to all the three planes of the image.

The stego images thus obtained were analyzed using measures like PSNR, MSE, R and SSIM. The algorithm achieved a maximum PSNR value of 87.5325, which is higher compared to the other established methodologies. Thus the proposed technique exhibits higher level of security and imperceptibility. The algorithm is implemented in the spatial domain to avoid the shortcomings of transform domain. Although the efficiency is higher for transform domain implementation, it suffers from a severe setback due to lower embedding capacity and higher computational cost. Hence, a spatial domain approach has been chosen.

Moreover, the generation of the unique key matrix randomizes the selection of the edge blocks required to embed the patient’s information. Moreover, the key matrix changes for every session. This enhances the security of the entire steganography method. The above arguments prove that the proposed technique is robust, secure and offers a fairly high embedding capacity.
